# Indigenous strengths-based approaches to healthcare and health professions education – Recognising the value of Elders’ teachings

**DOI:** 10.1177/00178969221088921

**Published:** 2022-04-07

**Authors:** Andrea Kennedy, Anika Sehgal, Joanna Szabo, Katharine McGowan, Gabrielle Lindstrom, Pamela Roach, Lynden (Lindsay) Crowshoe, Cheryl Barnabe

**Affiliations:** a Faculty of Health, Community and Education, Mount Royal University, Calgary, AB, Canada; b Department of Community Health Sciences, Cumming School of Medicine, University of Calgary, Calgary, AB, Canada; c Bissett School of Business, Mount Royal University, Calgary, AB, Canada; d Taylor Institute for Teaching and Learning, University of Calgary, Calgary, AB, Canada; e Department of Family Medicine, Cumming School of Medicine, University of Calgary, Calgary, AB, Canada; f Department of Medicine, Cumming School of Medicine, University of Calgary, Calgary, AB, Canada

**Keywords:** Indigenous, healthcare, health education, Indigenous Elders, strengths-based

## Abstract

**Background:**

A strengths-based lens is essential for the pursuit of health equity among Indigenous populations. However, health professionals are often taught and supported in practice via deficit-based approaches that perpetuate inequity for Indigenous peoples. Deficit narratives in healthcare and health education are reproduced through practices and policies that ignore Indigenous strengths, disregard human rights, and reproduce structural inequalities. When strengths are recognised it is possible to build capacities and address challenges, while not losing sight of the structural factors impacting Indigenous peoples’ health.

**Objective:**

In this paper, we examine Indigenous strengths-based approaches to policy and practice in healthcare and health professions education when delivered alongside teachings shared by Elders from the Cree, Blackfoot and Métis Nations of Alberta, Canada.

**Method:**

Literature and Elders’ teachings were used to shift strengths-based approaches from Western descriptions of what might be done, to concrete actions aligned with Indigenous ways.

**Results:**

Four pointers for future action adopting a strengths-based approach are identified: enacting gifts – focusing on positive attributes; upholding relationality – centring good relationships; honouring legacy – restoring self-determination; and reconciling truth – attending to structural determinants of health.

**Conclusion:**

Identified directions and actionable strategies offer a promising means to advance Indigenous health equity through strengths-based actions that change existing narratives and advance health equity.

*‘What comes out of our mouth either heals or hurts people*’.

*Teaching from Elder Tom Crane Bear shared by Elder Roy Bear Chief (Siksika Nation)*

## Positionality

The authors of this paper have Cree, Blackfoot, Métis and Settler ancestries, and all share a central commitment to good relations with Indigenous peoples. Our aim in this paper is to highlight opportunities for transformation and change in healthcare systems and health professions education in partnership with Indigenous peoples to advance health equity through the use of strengths-based approaches to Indigenous health.

## Introduction

Strengths-based approaches to health provide a ‘conceptual framework for approaching development and intervention . . . to counter both implicit and explicit deficit . . . [towards] solutions or opportunities that facilitate growth and thriving’ (Fogarty et al., 2018: 8). A strengths-based approach is essential for the pursuit of health equity with Indigenous peoples, yet a deficit-based approach is more frequently present in health professions education and practice. Recognising how discourse shapes reality through ‘systems of thoughts composed of ideas, attitudes, courses of actions, beliefs and practices’ (Foucault, 1972, as cited in Lessa, 2006: 285), deficit narratives concerning people, practices and ways of life perpetuate the use of deficit-based approaches to healthcare (Fforde et al., 2013).

Deficit-based communications and processes highlight the ‘lack of’ something and aim to identify failures and insufficiencies to be remedied through healthcare intervention. This, in turn, influences the behaviour of healthcare professionals, providers and educators who focus on loss and shortfalls (Fforde et al., 2013). Fundamental assumptions and the societal reinforcement of deficits (Hyett et al., 2019) facilitate harmful beliefs and practices, unfairly blaming Indigenous peoples for poor health outcomes rooted in broader historical and societal factors (Crowshoe et al., 2019; Jones et al., 2019).

Deficit narratives are pervasive in health communication, policy and practice, leading to poor health outcomes (Allan and Smylie, 2015) and reproducing system inequalities (Fogarty et al., 2018). Health professional education often takes place using a deficit model that protects systems inequalities and perpetuates Indigenous peoples’ experiences of ‘racism, marginalisation and exclusion’ (Allan and Smylie, 2015: 2). Given prolific Indigenous-specific racism, healthcare providers and health professions educators have a duty to respectfully engage with Indigenous peoples and transform research and teaching. In particular, there is a need to resist deficit-based narratives while promoting attributes and capacities within individuals and communities, to foster a strengths-based pathway towards reconciliation.

### Health as a human right for all

As authors and healthcare professionals, we respect Indigenous teachings that ‘we are all relatives’ (Deloria, 1999: 32), and recognise that health is a human right for all (World Health Organization [WHO], 2017). The United Nations Declaration of Rights for Indigenous Peoples (UNDRIP) outlines ‘minimum standards for the survival, dignity, and well-being of the Indigenous peoples of the world’ (UN, 2007: 28), and reflects on how inequity may be addressed through the protection of culturally based strengths, including the right to self-determination. Worldwide, the UNDRIP seeks to protect the rights of Indigenous peoples and, in Canada, is the guiding framework for the Truth and Reconciliation Commission of Canada’s (TRC, 2015b) Calls to Action. The TRC urges all Canadians to understand the truth of residential schools and work together to address the harm they caused, and repair relationships with Indigenous peoples. The implementation of UNDRIP is supported by the National Inquiry into Missing and Murdered Indigenous Women and Girls (MMIWG, 2019). The MMIWG inquiry examined the systemic forms of violence enacted against Indigenous women and girls, resulting in Calls for Justice, several of which have relevance to healthcare and for health educators. This report explicitly called for strengths-based approaches to research and reporting. It also stressed the importance of reclaiming cultural strengths and recognising deep-seated capacities for resilience, healing and wellness in individuals, families and communities. Such change is supported through *cultural security* and the commitment that health systems will not compromise the cultural rights, values, views and expectations of Indigenous peoples, as a rights-based policy commitment to engaging with cultural humility and facilitating culturally safe and quality healthcare (Lock et al., 2019).

### Intellectual and cultural humility

While there is rich Westernised evidence on the value of strengths-based approaches, there is an important opportunity to respect the self-determination of Indigenous peoples in defining such strengths from their perspective (Askew et al., 2020). This highlights the need to refocus our approach to strengths-based approaches with intellectual humility, by ‘recognizing the limits of one’s knowledge and appreciating others’ intellectual strengths’ (Porter and Schumann, 2018, p.139). It also encourages respect for Indigenous perspectives by embracing cultural humility as:

‘a process of self-reflection to understand personal and systemic biases and to develop and maintain respectful processes and relationships based on mutual trust. Cultural humility involves humbly acknowledging oneself as a learner when it comes to understanding another’s experience’ (First Nations Health Authority [FNHA], 2021).

Recognising the importance of intellectual and cultural humility, this paper seeks to extend understanding of Indigenous perspectives of strengths-based approaches in healthcare and health professions education to promote health equity. We humbly acknowledge how our prior understanding of Indigenous strengths-based approaches was incomplete, so we requested the guidance of three highly respected community Elders to help us.

### Aligning literature with Elders’ teachings

We began this process by seeking guidance from Elders in Alberta, Canada, while following local protocol with tobacco and gifting. The Elders asked that the literature review we had undertaken be shared on paper before introducing their teachings; they hoped this structure would create a bridge of understanding between Western and Indigenous ways of knowing, while also holding the Elders’ teachings with highest respect. Grandmother Doreen from Saddle Lake Cree Nation acknowledged that the academy involves ‘learning how to listen and learn with Elders’. She stressed the importance of ‘starting where people are at and bringing them forward with love and respect to learn our ways for reconciliation . . . we would never have a university without a library, and Elders are the library for Indigenous peoples’. We invite you to this co-learning space to bridge understanding by starting with a review of the literature followed by connections with Elders teachings.

### Literature review

An initial exploration of literature was undertaken to address the topic of strengths-based Indigenous health, while engaging in concurrent consultations with Indigenous Elders from Cree, Blackfoot, and Métis Nations of Alberta, Canada. This approach sought respectful engagement with local, placed-based Indigenous knowledge given our focus on Indigenous health in Alberta, Canada (Battiste, 2017). Knowledges and perspectives were connected using relationality (Deloria, 1999) as a framework for embodied Indigenous health. Equity was central to the approach given that ‘we are all relatives’ (Deloria, 1999: 32) while collective cultural strengths were brought to the fore by appreciating the dynamic relationship between individuals, communities, and all beings (Rountree and Smith, 2016). Analyses focused on interconnections of the whole rather than the use of a reductionist approach (Deloria, 1999; Kennedy et al, 2021b). Relational learning with Elders was supported through the use of a conversational method (Kovach, 2010) to respect oral tradition and the ‘deep purpose of sharing a story as a means to assist others’ (Kovach, 2010: 40).

Using the guiding question: ‘How might we shift towards an Indigenous strengths-based approach in healthcare and health professions education to advance Indigenous health equity?’ a purposive literature review was undertaken to include sources that were Indigenous co-led and included perspectives on topics pertaining to Indigenous health, equity, health education, strengths- and deficit-based approaches, resiliency, and institutional guidelines related to Indigenous healthcare and health education (particularly in the fields of nursing and medicine). The author team searched the profiles of each author listed on the selected articles included to determine Indigeneity. While the choice of literature was initially focused on Alberta, Canada, where the author team is located, it was later extended to include other Canadian and global resources. Selected quality sources were examined for issues relevant to Indigenous perspectives on strengths-based actions for health and well-being. A thematic analysis based on relationality (Deloria, 1999; Kovach, 2010) was conducted iteratively by the author team to identify common themes and patterns across included articles.

Since Western thought dominates healthcare and health professions education – and published Indigenous scholarship often adopts Western norms – our intention was to disrupt the current epistemic bias and re-centre Indigenous ways of knowing by examining Indigenous strengths as embodied by highly respected Indigenous knowledge holders through oral tradition. Elders are respected as the most valued knowledge holders who, through rigorous lifelong ceremonial training and practice, hold expert knowledge of Indigenous ways of being and knowing. Recognising how their contribution was key to the success of this process, we asked Elders for guidance on how we might understand Indigenous strengths. Their teachings are shared as stories aligning with Indigenous oral traditions (Kovach, 2010), and each Elder involved in the process approved this paper.

### Strengths-based approaches to Indigenous healthcare

Strengths-based approaches aim to support equitable opportunities and experiences in healthcare (see Table 1) and have been explored and defined from Indigenous perspectives of relationality within individuals and communities. Engaging in health-related activities focused on cultural attributes and resilience is associated with meaningful engagement (Cooper and Driedger, 2018) and positive health outcomes (Antonio et al., 2020; Kirmayer et al., 2011). To address the view that strengths-based approaches are vaguely defined (First Nations Information Governance Centre [FNIGC], 2020), findings from Bryant et al. (2021) are helpful in identifying three main strengths-based approaches: ‘resilience’ approaches related to personal skills, ‘socio-ecological’ approaches related to environmental context, and, ‘socio-cultural’ approaches related to social context including identity and cultural practices. Of interest, ‘resilience’ and ‘socio-ecological’ approaches are often construed as Westernised and individualistic, while the collectivist relationality of the ‘socio-cultural’ approach is more aligned with Indigenous ways of knowing.

**Table 1.** Strengths-based approaches to Indigenous healthcare.

Information source	Summary
1. Alberta Health Services Indigenous Health Transformational Roadmap (AHS, 2018) Location: Alberta, Canada	Summary: Strengths-based approaches in this roadmap are noted across guiding principles: embrace traditional knowledge and practices, distinguish and realise Indigenous peoples’ health care rights, know the distinct care needs of Indigenous peoples, empower self-determination and exercise reconciliation.
2. Conceptualisations of Health and Resilience among Native Hawaiians Antonio et al. (2020) Location: Hawaii, USA	Summary: This study found that strengths-based approaches have a positive impact on health outcomes and that Indigenous health is a state achieved through balance.
3. Strengths-based approaches to Indigenous public health Askew et al. (2020) Location: Australia	Summary: ‘A way of being’ is perspective taking of the community’s strengths, ‘A way of doing’ is engaging in meaningful relational practice and ‘A way of knowing’ is actively resisting racialising practices while respectfully engaging with Indigenous ways of knowing.
4. Strengths-based approaches in Indigenous health research Bryant et al. (2021) Location: Australia, Canada, United Kingdom	Summary: Three main approaches to strengths-based Indigenous health research include ‘resilience’ related to personal skills, ‘social–ecological’ related to structural environmental context and ‘sociocultural’ related to social context including identity and cultural practices.
5. The Indigenous Primary Health Care and Policy Research (IPHCPR) Network (Crowshoe et al., 2021) Location: Alberta, Canada	Summary: Derived principles from stakeholder engagement in Alberta include community-based research, strengths-based focus, Indigenous knowledges and ethics and having measurement aligned with Indigenous knowledge and derived from community.
6. Strengths-based approaches to knowledge translation within Indigenous health research (Cooper and Driedger, 2018,) Location: Canada	Summary: This study focused on the development of health research knowledge translation products in collaboration with Indigenous communities in Manitoba, Canada. Strengths-based approaches were aligned with a focus on attributes and respectful engagement of Indigenous knowledges.
7. First Nations Health Authority Summary Service Plan (FNHA, 2020) Location: British Columbia, Canada	Summary: Priorities identified in this service plan include having a First Nations Health Authority operating model and renewed partnerships with Nations. This service plan focuses on wellness, knowledge development and exchange, cultural humility and cultural safety, service excellence, leadership, and culture development.
8. Strengths-Based Approaches to Indigenous Research and the Development of Well-Being Indicators (FNIGC, 2020) Location: Canada	Summary: Recognising that there are gaps for measuring Indigenous strengths-based concepts, there is a need for systematic investigation of constructs. This can be done through respectful engagement with local protocols and knowledges, while reframing evidence quality from Indigenous perspectives.
9. Advancing Indigenous primary health care policy in Alberta, Canada (Henderson et al., 2018) Location: Alberta, Canada	Summary: Identified policy pathways from stakeholder engagement in Alberta included governance, Indigenous representation in health services leadership, adequate and flexible funding, continuous corridors of care based on communities and treaties, respecting Elders, and strengthening community resilience and capacity.
10. Rethinking resilience from Indigenous perspectives (Kirmayer et al., 2011) Location: Canada	Summary: In this summary of ‘Roots of Resilience’ with Mi’kmaq, Mohawk, Métis, and Inuit peoples, factors promoting resilience include components of person/identity, land, history, politics, languages, traditions, agency and activism.

The provincial health authority, Alberta Health Services (AHS) strives to advance a strengths-based approaches to Indigenous health through its ‘Indigenous Health Transformational Roadmap (AHS, 2018)’. Based on UNDRIP (UN, 2007), this identified six guiding principles to improve the health and wellness of Indigenous peoples based on the TRC (2015a), ethical space (Ermine, 2007), and Etuaptmumk/two-eyed seeing (Marshall et al., 2015). The Alberta Indigenous Primary Health Care and Policy Research (IPHCPR) Network, funded by the Canadian Institutes of Health Research (CIHR), aims to improve health outcomes among Indigenous peoples (Crowshoe et al., 2021; Henderson et al., 2018). Central to the IPHCPR Network’s work is a ‘strengths-based lens focused on resilience’ which is seem as ‘essential for the pursuit of health equity with Indigenous populations’ (Crowshoe et al., 2021: 729). Work is supported upstream by the CIHR funded Alberta Indigenous Mentorship in Health Innovation (AIM-HI) Network’s commitment to both Indigenous and non-Indigenous health researchers and community partners, to strengthen and provide new opportunities for First Nations, Métis, and Inuit mentees to pursue health research careers.

The First Nations Health Authority (FNHA) has led Indigenous governance in healthcare in collaboration with provincial partners in British Columbia, Canada, since 2013 (FNHA, 2020). Seeing culture as a shared value and strength, FNHA Indigenous community-driven and nation-based approaches are driving reform and innovation for health equity with a focus on capacity building and self-determination for holistic wellness. Shared values and key directives support priority goals including health governance, Indigenous perspectives, health programme and service excellence and operational excellence (FNHA, 2020: 15).

### Strengths-based approaches for health professions education

Strengths-based approaches are present in some frameworks for Indigenous health professions education, especially in nursing and medicine (see Table 2). Healthcare and health professions education informed by such frameworks aim for health equity through the use of Indigenous perspectives on well-being, human dignity, capacity building, agency and resilience (Crowshoe et al., 2019; Jones et al., 2019; Kennedy et al., 2021a; Lindstrom, 2018).

**Table 2.** Strengths-based approaches to Indigenous health professions education.

Information source	Summary
11. Association of Faculties of Medicine Canada (AFMC, 2019) Location: Canada	Summary: Indicators of response to the TRC Calls to Action within medical education curricula include promoting Indigenous community relationships (rights-based approaches and social accountability), and fostering learning environments (invest in Indigenous faculty and staff with infrastructure).
12. Aboriginal Nurses Association of Canada (ANAC, 2009) Location: Canada	Summary: Core nursing education competencies to promote Indigenous representation in nursing education include postcolonial understanding, communication, inclusivity, respect, Indigenous knowledge and mentoring, and supporting students for success.
13. College and Association of Registered Nurses of Alberta (CARNA, 2020) Location: Alberta, Canada	Summary: Indigenous oral teachings and visual artwork is presented as a means to support reconciliation through compassionate and trauma-informed nursing care with Indigenous peoples.
14. Canadian Association of Schools of Nursing (CASN, 2013) Location: Canada	Summary: This framework suggests bringing Indigenous culture, history, and context, and creating safe and supportive classroom environments to address cultural safety and the representation Indigenous students in nursing education.
15. Canadian Association of Schools of Nursing (CASN, 2020) Location: Canada	Summary: A framework to support decolonisation, Indigenisation, and reconciliation in nursing education through strategies including foundational supports for reconciliation and the recruitment and retention of Indigenous students.
16. College of Family Physicians of Canada–CanMEDS-FM Indigenous Health Supplement (CFPC, 2020) Location: Canada	Summary: Document presenting competencies to support healthcare providers and educators to better engage in care that respects inherent strengths within the culture and history of Indigenous peoples to promote culturally safe care.
17. Indigenous Physicians Association of Canada and The Royal College of Physicians and Surgeons of Canada (IPAC and RCPSC, 2009) Location: Canada	Summary: Core competencies defined through roles including the medical expert who provides culturally-safe and relational care, and the communicator who establishes positive and therapeutic relationships with Indigenous peoples.
18. Educating for indigenous health equity (Jones et al., 2019) Location: New Zealand, Canada, Hawaii-United States, Australia	Summary: In this consensus statement for Indigenous health equity, key principles for medical education included: colonisation and decolonisation processes, advocacy for Indigenous rights and health equity, and responsibilities for faculty, students and health education and healthcare institutions.
19. Reconciling taking the ‘Indian’ out of the nurse (Kennedy et al., 2021a) Location: Canada	Summary: In this conceptual paper, pathways to mitigate the ongoing impact of assimilation in nursing education are discussed. It was determined that cultural humility is a means to advance cultural safety along with respectful engagement with Indigenous knowledges.
20. Trauma and resilience in Aboriginal adult learners’ post-secondary experience (Lindstrom, 2018) Location: Canada	Summary: In this ethnographic study, it was found that trauma, poverty, lateral violence, and inequality are factors influencing post-secondary students. Post-secondary educators have an opportunity to support learners to engage through a pedagogy of resilience, noting oppression and power in post-secondary education.

In 2009, frameworks with core competencies were developed by the Aboriginal Nurses Association of Canada (ANAC) (now the Canadian Indigenous Nurses Association, CINA) and the Indigenous Physicians Association of Canada (IPAC), drawing attention to the importance of cultural safety, relational ethics and respectful engagement with Indigenous knowledges and communities. Strengths-based competencies include the ability to ‘critically appraise the strengths and limitations of available data used as key indicators of Canadian Aboriginal Health’ (ANAC, 2009: 13; IPAC and the Royal College of Physicians and Surgeons of Canada, 2009: 16). More recently, the College of Family Physicians of Canada (CFPC, 2020) has outlined specific physician roles and key competencies. Within this context, strengths-based approaches and outcomes are recognised as motivating leverage for holistic wellbeing including ‘healing-centred engagement’ (CFPC, 2020: 7), trauma-informed care and health advocacy to support culturally safe care (CFPC, 2020).

The Canadian Association of Schools of Nursing (CASN) in collaboration with CINA has developed a framework to address the social, cultural and contextual determinants of health with Indigenous peoples throughout nurse education in culturally safe classrooms with ‘recognition and support of client and community strengths (CASN, 2013: 11)’. It has also developed the ‘Framework of Strategies for Nursing Education to Respond to the Calls to Action of Canada’s Truth and Reconciliation Commission (CASN, 2020)’. The College and Association of Registered Nurses of Alberta (CARNA) has released an online certificate ‘Stronger Together: Learning through Indigenous Perspectives’ to advance reconciliation in nursing clinical practice, administration, education and research (CARNA, 2020). In addition, the Association of Faculties of Medicine Canada (AFMC) has investigated the Truth and Reconciliation Commission’s Calls to Action #23 and #24 and provided curriculum action statements and indicators relevant to medical education (AFMC, 2019).

**Figure 1.**
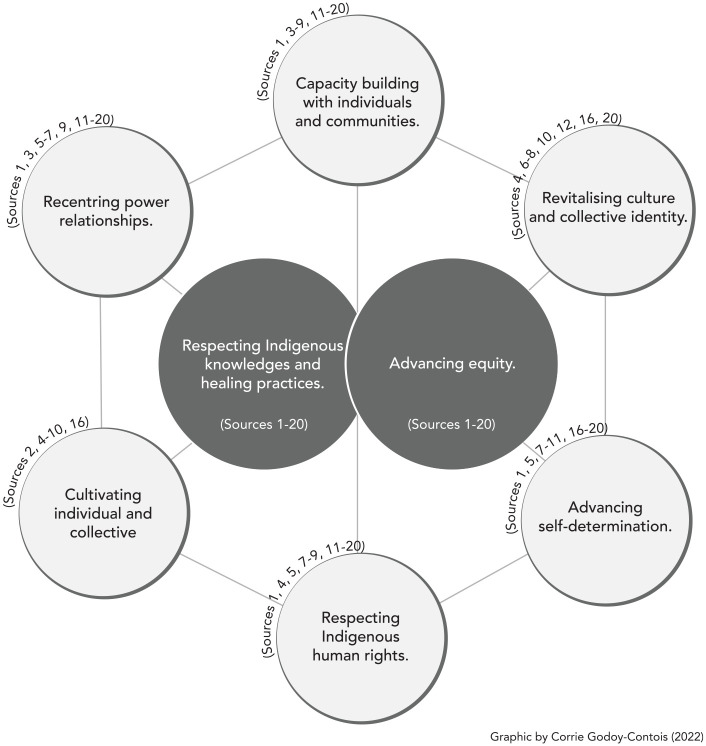
Strengths-based actions identified in sources.

Strengths-based approaches in healthcare and health professions education are reflected in the eight interconnected actions to support Indigenous health equity shown in Figure 1. These include (1) respecting Indigenous human rights, (2) respecting Indigenous knowledges and healing practices, (3) re-centring power relationships, (4) revitalising culture and collective identity, (5) cultivating individual and collective resilience, (6) advancing equity, (7) advancing self-determination and (8) capacity building with individuals and communities. Similar to Askew et al. (2020), these ways of defining strengths-based approaches actively resist oppression while offering a decolonial embodied interpretation aligned with dynamic relational Indigenous worldviews.

In our review of the literature, we found that author’s positionality was not commonly stated. Without an indication about positionality, it is not possible to understand an author’s place and purpose. Western academic practices typically overlook the importance of positionality in research, thereby marginalising Indigenous expertise and the relational context.

### Indigenous strengths-based actions identified by Elders

#### Elder Grandmother Doreen Spence (Saddle Lake Cree Nation)

Strengths are about a way of being through Relationships with ourselves, people, the land, ancestors and all beings; these connections are a reflection and extension of self. These strengths are lived through actions in the Circle as opportunities to engage, learn, connect and re-engage when we lose our way. Strengths are directed at the Common Good, for the benefit of all, and validated in our Accountability to the common good of all beings. We share our strengths with Humility and Respect, and Without Fear. This is practiced as Helping and understood as a Present opportunity. We need to understand and share strengths as Gifts, and it is our Responsibility to keep that vibration in our hearts.

I share a concern held by many–that Indigenous peoples are talked about negatively–so much so that we accept this as ‘normal’ yet this has a negative impact on the health and well-being of individuals and communities. I agree with the term ‘deficit discourse’ because this represents what Indigenous peoples face in statistics, news and everyday interactions. These deficits do not fully represent who we are. We need to be respected as human beings–no one above or below another. For instance, our medicines and ways were used to help the Settlers to survive when they arrived–where is this history? We have a rich legacy of strengths that continues today. Strengths are not a list of things to achieve, strengths are based on our natural laws on how we need to live in a good way.

Through her words above, Grandmother teaches us to understand and share strengths as gifts to be lived out through relationships with each other and all beings. While the term ‘strengths’ may be a noun, Grandmother’s teachings redirect our understanding to Indigenous ways of knowing by animating strengths as actionable concepts acquired and developed through lifelong learning. This redirection is important since it calls us to understand strengths from Indigenous perspectives. Strengths are gifts to be shared for the greater good, and all people hold this opportunity and responsibility as helpers, noting the legacy of Indigenous peoples who helped Settlers to survive.

#### Elder Roy Bear Chief (Blackfoot, Siksika Nation)

Our strengths are in our actions – when we look, listen and learn. Strengths are not things – strengths are the good actions of how we live and treat each other. We are born into culture. This is our responsibility and birthright to take care of it. We need to understand the way of life. This starts with language – to speak the language is to understand the culture; to understand the culture is to understand the way of life. We need to embody this. We have a responsibility to teach this to the young people. We need to listen, especially to the old ones. Each Elder has their own repository to unlock and share. We need to make space for Elders to open up their bundle to share what they have retained over the years. If we don’t respect this, we will destroy this knowledge. The beauty of listening and then explaining is the gift of sharing knowledge. We need to see where this comes from and sprinkle culture back into our landscape like seeds that will grow. We can all be helpers.

Two-eyed seeing helps us heal the losses of colonisation. We are stuck in two worlds and can reclaim our culture by sharing our culture with others. We need to create relevance in the institutional landscape by sharing stories and helping others pick up the teachings so that they have meaning in the work we do together. Two-eyed seeing branches out like a fork, and helps us decide which lens to use and why. We have to be true to the lenses we use to raise awareness, create relationships and move to understanding. Then we can deconstruct the past together and understand where we can help. We all need two-eyed seeing or we are stuck in our own perspective – this is beyond Indigenous/non-Indigenous ways – this is not about polarity, but rather about respect, humility, wisdom and responsibility. Our eyes are connected to our brains/minds, and this shapes how we hear, and ultimately how we communicate – we can help or hurt people with our words, based on how we move from misunderstood– understood – understand. We need to respect the traditional lands where we are living, working and raising our families. How do we sit in humility with traditional knowledge holders to develop understanding? We also have to take care of ourselves to be helpers in the health profession, and to honour human dignity and treat all people with kindness-compassion. We need to honour our traditional names and how this carries our ancestors forward. This is our purpose. We are born into culture and need to keep it going.

Elder Roy Bear Chief’s teaching further stresses how strengths are the good actions inherent in how we live and treat each other, noting our responsibility as helpers in accordance with our purpose. He shares how there is strength in creating space for sharing knowledges and creating connections to promote the building of relationships within institutions, guided by the principles of Etuaptmumk/two-eyed seeing. Strength lies exploring our capacity to develop understanding beyond our own perspective, it is to honour the lens that deserves respect and humility to find a space in the two worlds. Strength is leveraged by honouring language and developing a deeper understanding of one’s culture and place-based knowledge to understand and embody the way of life and its teachings as a legacy of cultural birthright and responsibility.

#### Elder Noreen McAteer (Métis, Northern Alberta-Fort Vermilion)

First, we need to learn to love each other: I love all people. No one is better than another. You have to respect all people to love yourself. Then you can fully embrace your identity. I respect the traditional ways and my Cree grandmother. I wouldn’t be Métis without my Scottish grandfather. You have to make your own place – take what is good from each side of your family and that is YOU. For some, being Métis was like being a second-class citizen, but I did not grow up with that. It does not help to put down one another. I love all of my relatives.

Next, it is important to always look for the good: there is good in everything and everyone. This is about the strength to focus on the good in all things including ourselves and each other and in different situations. We need to look for the good in our relationships with respect. We start by earning respect by living with good values and then we earn respect with our actions. Since this is all about relationships, we also need to give respect. You also need to respect your culture and understand how culture changes over time.

When you are always learning it means that you can also always be changing for the better, and still remembering where you come from and your purpose in life. Remember to share your gifts and respect the ceremonies. Be part of the community as a helper with the gifts that you have. Take good care of yourself so that you can help your family and community. Your strengths are these combined actions. No one can take this from you because your strengths are at the heart of you – you live them in your actions.

Elder Noreen McAteer teaches us to make our own place as strength resides in the ability to love and give respect to yourself, others and to culture and community as a whole. Strength lies in recognising and embracing change and finding one’s unique role and contribution to the community. Elder Noreen discusses dismantling stereotypes as a means of re-centring relationships. Strength lies in restoring Indigenous ways of being and fostering meaningful relationships within oneself and with the community.

Taken together, Elders’ teachings guide us on the use of strengths-based approaches as identified in the literature (Askew et al., 2020). Strengths are gifts that come alive in our actions as helpers for the greater good through relationships with all beings. Indigenous strengths reside in the capacity to love all of our relationships with humility, reciprocity, and respect. The legacy of Indigenous strengths is carried through generations and understood in place – the land and within our own selves – with love as the binding element that grounds us into ourselves and further allows us to value one another. This is how culture is created and shared: by enacting one’s gifts, upholding relationality as community helpers, and honouring cultural legacy. Indigenous health and health professions education could be well-positioned in reconciling with Indigenous peoples through these teachings. Truthfully reconciling disparities as the scaffolding of Indigenous strengths-based approaches enables us to move forward.

## Supportive actions to promote the use of strengths-based approaches

This exploration of the literature and Elders’ teachings on Indigenous strengths-based health emphasises the importance of healthcare providers being inclusive of Indigenous perspectives. Healthcare providers and educators are called upon to refocus on positive attributes and respectfully engage with Indigenous knowledges and peoples while reframing health disparities from a structural perspective as intimately linked to colonisation. Through an ethical space of engagement (Ermine, 2007), we can come to respect how Indigenous and Western views are disparate yet may be appreciated in convergence (Ray, 2012). Elder Roy Bear Chief utilises the metaphor of a fork to enable a process of Etuaptmumk/two-eyed seeing (Marshall et al., 2015) that feels protective, but with ‘just enough’ sharing and distance to offer what is needed. Healthcare providers and educators need to be prepared to respectfully co-facilitate an ethical space in which Indigenous wisdom can be braided into practice. Within this space of Etuaptmumk/two-eyed seeing and relationality, we offer a synthesis of key literature findings as shown in Figure 1, and Elders’ teachings that revealed important directions for healthcare providers and educators with supporting action strategies in Table 3 to move towards Indigenous strengths-based approaches.

**Table 3.** Indigenous strengths-based directions and action strategies for healthcare and health professions education.

Directions	Action strategies
Enacting Gifts: focusing on positive attributes that facilitate well-being with Indigenous peoples	• understanding Indigenous perspectives of strengths and holistic health • finding the good in health patterns, evidence and stories • valuing contributions of Indigenous peoples through communications, programmes and policies • developing indicators and identifying resources to support strengths-based approaches
Upholding Relationality: centering good relations with Indigenous peoples	• engaging in an ethical space with respect for the Principles of Reconciliation • disrupting racialising stereotypes • humanising interactions with dignity and respect • honouring collectivism and interconnection with people and all beings
Honouring Legacy: restoring self-determination with Indigenous peoples	• Implementing the United Nations Declaration of Human Rights for Indigenous Peoples • respecting Indigenous ways of being, knowing, doing and identity • recognising the vital role and contribution of Elders • decolonising discourse and power with meaningful opportunities for collaboration
Reconciling Truth: attending to structural determinants of health with Indigenous peoples	• exploring how historic and social contexts impact health behaviours and outcomes • engaging with cultural humility and structural competency for ongoing co-learning • facilitating critical learning and constructive actions to address root causes of health inequity • advancing health equity through access to Indigenous primary health care

### Direction 1: Strengths are gifts. Focusing on positive attributes that facilitate well-being with Indigenous peoples

Reflect on the Elders’ teachings that strengths are gifts in action, and engage in positive change knowing healthcare providers and health professions educators have the capacity as helpers to recognise and optimise Indigenous peoples’ potential for well-being. By resisting Western ideologies that marginalise Indigenous ways of knowing, and refocusing on Indigenous perspectives of positive attributes, we may better understand and facilitate the ‘good’ in Indigenous health rather than keeping a spotlight on health deficiencies and disparities. Through Etuaptmumk/two-eyed seeing grounded in cultural humility, it is possible to recognise strengths from an Indigenous perspective while respecting how this may differ from the Western approaches that currently dominate healthcare and health professions education.

### Direction 2: Uphold relationality. Centring good relations with Indigenous peoples

Reflect on the Elders’ teachings that strengths are leveraged through relationships, and engage in positive change knowing healthcare providers and health professions educators have the capacity to engage as co-learners in Etuaptmumk/two-eyed seeing. By working together, healthcare providers and educators can develop a shared understanding of strengths in an ethical space of engagement, and uphold relationality. Centring good relations with Indigenous peoples is a means to improve our interactions grounded on values of dignity and respect. By centring good relations with Indigenous peoples, we can honour the interconnectedness of people and all beings, and recognise their strengths.

### Direction 3: Honour legacy. Restoring self-determination with Indigenous peoples

Reflect and enact the Elders’ teachings that Indigenous peoples have a rich legacy of strengths, and that culture is a responsibility and a birthright. Engage in positive change knowing healthcare providers and health professions educators have the capacity to respect human rights for Indigenous self-determination ‘to freely pursue their economic, social and cultural development’ (UNDRIP, 2007: 3). This requires honouring Indigenous knowledge spheres, teaching and culture, including recognising the role and contribution of Elders to restore self-determination among Indigenous peoples. Honouring the legacy of Indigenous strengths, while critically acknowledging the drivers and dominance of Western health models, provides an opportunity to create meaningful avenues for work together to advance Indigenous health equity.

### Direction 4: Reconciling truth. Attending to structural determinants of health with Indigenous peoples

Reflect and act upon Elders’ teaching that Etuaptmumk/two-eyed seeing helps us heal the losses of colonisation, and engage in positive change knowing healthcare providers and health professions educators can promote changes in their institutions while honouring human dignity. Attending to the structural determinants of health requires an exploration and deeper understand of how historic and social factors continue to impact health behaviours and outcomes with Indigenous peoples. We may advance positive disruption by engaging in co-learning with cultural humility and structural humility – this includes understanding the limits of our influence as healthcare providers and educators, and the need for outcomes such as cultural safety and transformed health and education systems to support Indigenous wellbeing.

## Strengths and limitations

This paper has sought to bridge Western ways of research synthesis with Indigenous ways of seeing and doing, allowing an appreciation of different worldviews within a respectful and ethical space. The contributions of Elders as cherished knowledge holders are included here to show respect and to allow them have the final word. A limitation of the work, however, is that the directions and action strategies identified may be limited in generalisability, since only local Indigenous Elders from Alberta, Canada were invited to share their insights. Further respectful engagement is required with Indigenous knowledge holders in other settings. Another limitation derives from that fact that cited literature was selected purposefully rather than through a systematic, structured search. Finally, we were unable to locate evaluation techniques to demonstrate shifts in discourse and Indigenous health outcomes when a strengths-based approach such as that described here is implemented. Nonetheless, findings offer a step forward towards formalising recommendations for the use of Indigenous health strengths-based approaches.

## Conclusion

Indigenous perspectives on strengths-based approaches can help shift narratives and leverage collective strengths within Indigenous individuals and communities. We urge researchers and educators working on similar issues to identify their positionality and relationality within context, to advance intellectual and cultural humility along with equity in their published work, while making Indigenous authors’ contributions visible as a decolonial effort. The benefits of reviewing the literature alongside respectful engagement with Indigenous knowledge holders is advocated to establish deeper understanding. In this paper, exploring relevant literature alongside teaching by local Indigenous Elders gave rise to directions and action strategies to decolonise health systems and advance health equity.

## References

[bibr1-00178969221088921] Aboriginal Nurses Association of Canada (ANAC) (2009) Cultural competence and cultural safety in nursing education. ANAC. Available at: https://www.ontariomidwives.ca/sites/default/files/2019-08/Cultural%20Competence%20and%20Cultural%20Safety%20-%20Nursing%20ed.pdf

[bibr2-00178969221088921] Alberta Health Services Indigenous Health Transformational Roadmap (AHS) (2018) Population, public, and Indigenous health SCN. Available at: https://www.albertahealthservices.ca/assets/about/scn/ahs-scn-ppih-ih-roadmap.pdf

[bibr3-00178969221088921] Allan B Smylie J (2015) First peoples, second class treatment: The role of racism in the health and well-being of indigenous peoples in Canada. Discussion paper. Wellesley Institute. Available at: https://www.wellesleyinstitute.com/wp-content/uploads/2015/02/Summary-First-Peoples-Second-Class-Treatment-Final.pdf

[bibr4-00178969221088921] Antonio MC Keaulana S Chung-Do JJ , et al. (2020) (Re) constructing conceptualizations of health and resilience among native Hawaiians. Genealogy 4(1): 8.

[bibr5-00178969221088921] Askew DA Brady V Mukandi B , et al. (2020) Closing the gap between rhetoric and practice in strengths-based approaches to Indigenous public health: A qualitative study. Australian and New Zealand Journal of Public Health 44(2): 102–105. 31914233 10.1111/1753-6405.12953

[bibr6-00178969221088921] Association of Faculties of Medicine Canada (AFMC) (2019) Joint commitment to action on Indigenous health. AFMC. Available at: https://www.afmc.ca/sites/default/files/pdf/AFMC_Position_Paper_JCAIH_EN.pdf

[bibr7-00178969221088921] Battiste M (2017) Decolonizing Education: Nourishing the Learning Spirit. Vancouver, BC, Canada: UBC press.

[bibr8-00178969221088921] Bryant J Bolt R Botfield JR , et al. (2021) Beyond deficit: ‘strengths-based approaches’ in Indigenous health research. Sociology of Health & Illness 43: 1405–1421. 34145599 10.1111/1467-9566.13311

[bibr9-00178969221088921] Canadian Association of Schools of Nursing (CASN) (2013) Educating nurses to address socio-cultural, historical, and contextual determinants of health among Aboriginal peoples. Available at: https://casn.ca/wp-content/uploads/2014/12/ENAHHRIKnowledgeProductFINAL.pdf

[bibr10-00178969221088921] Canadian Association of Schools of Nursing (CASN) (2020) Framework of strategies for nursing education to respond to the calls to action of Canada’s Truth and Reconciliation Commission. Available at: https://www.casn.ca/2020/11/framework-of-strategies-for-nursing-education-to-respond-to-the-calls-to-action-of-canadas-truth-and-reconciliation-commission/

[bibr11-00178969221088921] College and Association of Registered Nurses of Alberta (CARNA) (2020) Stronger together: Learning through Indigenous perspectives. Certificate Course. Available at: https://nurses.ab.ca/

[bibr12-00178969221088921] College of Family Physicians of Canada (CFPC) (2020) CanMEDS-FM Indigenous health supplement. Available at: https://www.cfpc.ca/CFPC/media/PDF/CanMEDS-IndigenousHS-ENG-web.pdf

[bibr13-00178969221088921] Cooper E Driedger S (2018) Creative, strengths-based approaches to knowledge translation within indigenous health research. Public Health 163: 61–66. 30098469 10.1016/j.puhe.2018.06.020

[bibr14-00178969221088921] Crowshoe LL Henderson R Jacklin K , et al. (2019) Educating for equity care framework: Addressing social barriers of Indigenous patients with type 2 diabetes. Canadian Family Physician 65(1): 25–33. 30674510 PMC6347314

[bibr15-00178969221088921] Crowshoe LL Sehgal A Montesanti S , et al. (2021) The Indigenous primary health care and policy research network: Guiding innovation within primary health care with Indigenous peoples in Alberta. Health Policy 125(6): 725–731. 33685657 10.1016/j.healthpol.2021.02.007

[bibr16-00178969221088921] Deloria V (1999) Spirit & Reason: The Vine Deloria, Jr., Reader. Golden, CO: Fulcrum Publishing.

[bibr17-00178969221088921] Ermine W (2007) The ethical space of engagement. Indigenous Law Journal 6: 193.

[bibr18-00178969221088921] Fforde C Bamblett L Lovett R , et al. (2013) Discourse, deficit and identity: Aboriginality, the race paradigm and the language of representation in contemporary Australia. Media International Australia 149(1): 162–173.

[bibr19-00178969221088921] First Nations Health Authority (FNHA) (2020) 2020/2021 FNHA summary service plan. Available at: https://www.fnha.ca/about/news-and-events/news/summary-service-plan-2020-21-release

[bibr20-00178969221088921] First Nations Health Authority (FNHA) (2021) Cultural safety and humility. Available at: https://www.fnha.ca/wellness/wellness-and-the-first-nations-health-authority/cultural-safety-and-humility 10.12927/hcq.2024.27429 39492739

[bibr21-00178969221088921] First Nations Information Governance Centre (FNIGC) (2020) Strengths-based approaches to Indigenous research and the development of well-being indicators. Available at: https://fnigc.ca/wp-content/uploads/2021/05/FNIGC-Research-Series-SBA_v04.pdf

[bibr22-00178969221088921] Fogarty W Lovell M Heron MJ , et al. (2018) Deficit discourse and strengths-based approaches: Changing the narrative of Aboriginal and Torres Strait Islander health and wellbeing. National Centre for Indigenous Studies, The Australian National University. Available at: https://www.lowitja.org.au/content/Document/Lowitja-Publishing/deficit-discourse-strengths-based.pdf

[bibr23-00178969221088921] Henderson R Montesani S Crowshoe L , et al. (2018) Advancing Indigenous primary health care policy in Alberta, Canada. Health Policy 122(6): 638–644. 29751973 10.1016/j.healthpol.2018.04.014

[bibr24-00178969221088921] Hyett SL Gabel C Marierrison S , et al. (2019) Deficit-based Indigenous health research and the stereotyping of Indigenous Peoples. Canadian Journal of Bioethics 2(2): 102–109.

[bibr25-00178969221088921] The Indigenous Physicians Association of Canada and the Royal College of Physicians and Surgeons of Canada (2009) First Nations, Inuit and Métis Health Core Competencies for Continuing Medical Education; Winnipeg & Ottawa. Available at: https://www.hhr-rhs.ca/index.php?option=com_mtree&task=viewlink&link_id=10851&Itemid=109&lang=en

[bibr26-00178969221088921] Jones R Crowshoe L Reid P , et al. (2019) Educating for indigenous health equity: An international consensus statement. Academic Medicine 94(4): 512. 30277958 10.1097/ACM.0000000000002476 PMC6445615

[bibr27-00178969221088921] Kennedy A Bourque DH Bourque DE , et al. (2021a) Reconciling taking the ‘Indian’ out of the nurse. Quality Advancement in Nursing Education 7(1): 1–16.

[bibr28-00178969221088921] Kennedy A McGowan K Lindstrom G , et al. (2021b) Relational learning with Indigenous communities: Elders’ and Students’ perspectives on reconciling Indigenous service-learning. International Journal of Research on Service-learning and Community Engagement 8(1): 1–16.

[bibr29-00178969221088921] Kirmayer LJ Dandeneau S Marshall E , et al. (2011) Rethinking resilience from Indigenous perspectives. Canadian Journal of Psychiatry 56(2): 84–91. 21333035 10.1177/070674371105600203

[bibr30-00178969221088921] Kovach M (2010) Conversation method in Indigenous research. First Peoples Child & Family Review: An Interdisciplinary Journal Honouring the Voices, Perspectives, and Knowledges of First Peoples through Research, Critical Analyses, Stories, Standpoints and Media Reviews 5(1): 40–48.

[bibr31-00178969221088921] Lessa I (2006) Discursive struggles within social welfare: Restaging teen motherhood. British Journal of Social Work 36(2): 283–298.

[bibr32-00178969221088921] Lindstrom GE (2018) Trauma and resilience in aboriginal adult learners’ post-secondary experience. PhD Thesis, University of Calgary, Calgary, AB, Canada.

[bibr33-00178969221088921] Lock MJ Hartz D Martin R , et al. (2019) An Aboriginal Cultural Safety and Security Framework: Improving Aboriginal Health Outcomes through Culturally Safe and Secure Mainstream Healthcare Governance and Practice. Port Macquarie, NSW, Australia: Mid North Coast Local Health District and the Mid North Coast Aboriginal Health Authority.

[bibr34-00178969221088921] Marshall M Marshall A Bartlett C (2015) Two-eyed seeing in medicine. In: Greenwood M de Leeuw S Lindsay NM (eds) Determinants of Indigenous Peoples’ Health in Canada: Beyond the Social. Toronto, ON, Canada: Canadian Scholars’ Press, pp. 16–24.

[bibr35-00178969221088921] National Inquiry into Missing and Murdered Indigenous Women and Girls (MMIWG) (2019) Reclaiming power and place: The Final Report of the National inquiry into missing and murdered Indigenous women and girls: Calls for justice. Available at: https://www.mmiwg-ffada.ca/wp-content/uploads/2019/06/Final_Report_Vol_1b.pdf

[bibr36-00178969221088921] Porter T Schumann K (2018) Intellectual humility and openness to the opposing view. Self and Identity 17(2): 139–162.

[bibr37-00178969221088921] Ray LWG (2012) Deciphering the ‘Indigenous’ in Indigenous methodologies. AlterNative: An International Journal of Indigenous Peoples 8(1): 85–98.

[bibr38-00178969221088921] Rountree J Smith A (2016) Strength-based well-being indicators for Indigenous children and families: A literature review of Indigenous communities’ identified well-being indicators. American Indian Alaska Native Mental Health Research 23(3): 206–220. 27383093 10.5820/aian.2303.2016.206

[bibr39-00178969221088921] Truth and Reconciliation Commission of Canada (2015a) Calls to action. Available at: http://trc.ca/assets/pdf/Calls_to_Action_English2.pdf

[bibr40-00178969221088921] Truth and Reconciliation Commission of Canada (2015b) Final report. Available at: https://publications.gc.ca/collections/collection_2015/trc/IR4-7-2015-eng.pdf

[bibr41-00178969221088921] United Nations (2007) Declaration on the Rights of Indigenous Peoples (UNDRIP). Available at: http://www.un.org/esa/socdev/unpfii/documents/DRIPS_en.pdf 10.1016/S0140-6736(07)61742-5 18037079

[bibr42-00178969221088921] World Health Organization (WHO) (2017) Health is a fundamental human right. Available at: https://www.who.int/news-room/commentaries/detail/health-is-a-fundamental-human-right

